# Re‐Evaluating the Role of Statistical Learning: Domain‐General Predictors of Language in Typical and Atypical Development

**DOI:** 10.1111/cogs.70261

**Published:** 2026-09-09

**Authors:** Krisztina Sára Lukics, Dorottya Dobó, Péter Rácz, Ágnes Lukács

**Affiliations:** ^1^ Department of Cognitive Science Faculty of Natural Sciences Budapest University of Technology and Economics, Muegyetem rkp. 3., H‐1111 Budapest Hungary

**Keywords:** Statistical learning, Working memory, Vocabulary, Grammatical abilities, Dimensional perspective, Developmental language disorder, Autism spectrum disorder, Attention deficit hyperactivity disorder

## Abstract

The ability to detect statistical regularities in the input is widely assumed to support language acquisition, but its unique contribution to individual differences in language outcomes remains uncertain, especially in neurodevelopmental conditions. This study tested whether statistical learning predicts expressive vocabulary and grammatical skills in children with typical development and in groups with developmental language disorder, autism spectrum disorder or attention deficit hyperactivity disorder. A total of 102 Hungarian‐speaking children aged 8 to 15 years completed tasks assessing statistical learning, auditory and visual perceptual speed, short‐term and working memory, sentence repetition, and expressive vocabulary. Statistical learning showed modest associations with language measures, but the effects of perceptual speed and working memory were stronger. Verbal working memory consistently emerged as the strongest predictor of vocabulary and sentence repetition across all groups. At the same time, the relatively low reliability of the statistical learning measures may have attenuated the observed associations, potentially leading to an underestimation of the strength of their relationship with language. The pattern of associations did not differ systematically between diagnostic groups, consistent with the idea that variation in language outcomes reflects general cognitive differences that cut across traditional diagnostic categories. This finding is consistent with dimensional accounts of neurodevelopment, which propose that differences between conditions arise through quantitative rather than qualitative variation in core cognitive processes. However, the various dimensions were hard to disentangle at this sample size. By demonstrating this pattern for language abilities, the present study extends the dimensional perspective to the domain of language development and highlights the importance of accounting for shared cognitive mechanisms when studying typical and atypical language variation.

## 1 Introduction

Language is characterized by structured patterns and regularities at multiple levels. Despite its complexity, linguistic knowledge can emerge gradually from simple underlying learning mechanisms (Tomasello, 2010). Statistical learning, defined as the ability to implicitly detect environmental regularities based on frequency distributions and transitional probabilities (Aslin, 2017; Conway, 2020; Frost, Armstrong, Siegelman, & Christiansen, 2015), is a fundamental mechanism supporting language acquisition. Studies have shown that statistical learning can explain core processes involved in language learning. For example, infants and adults can segment a novel continuous speech stream into words based solely on the transitional probabilities between syllables (Saffran, Aslin, & Newport, 1996; Saffran, Newport, & Aslin, 1996), and they can map word–referent pairings based on their probabilistic co‐occurrences across situations (Smith & Yu, 2008; Yu & Smith, 2007). Recent advances in large language models further support the idea of language acquisition through simple learning mechanisms by demonstrating that large‐scale language systems can emerge purely through sensitivity to probabilistic relationships between linguistic elements (Contreras Kallens, Kristensen‐McLachlan, & Christiansen, 2023).

The role of statistical learning in language acquisition is further supported by evidence showing associations between individual differences in statistical learning and a range of language abilities. Studies report significant correlations between statistical learning and speech perception in noise (Conway, Bauernschmidt, Huang, & Pisoni, 2010; Conway, Karpicke, & Pisoni, 2007), lexical abilities (Mainela‐Arnold & Evans, 2014; Smith & Yu, 2008), adult natural language proficiency (Daltrozzo et al., 2017), syntactic priming in preschoolers (Kidd, 2012), broad linguistic abilities in primary school children (Kidd, Arciuli, Christiansen, & Smithson, 2023), syntax comprehension (Kidd & Arciuli, 2016; Misyak & Christiansen, 2012; Torkildsen, Arciuli, & Wie, 2019), semantic processing of sentences (Munding, Forgács, Lukics, & Lukács, 2025), sensitivity to frequency distributions in natural language (Isbilen, McCauley, & Christiansen, 2022), and reading development in typical readers (Apfelbaum, Hazeltine, & McMurray, 2013; Arciuli & Simpson, 2012; Spencer, Kaschak, Jones, & Lonigan, 2015). A recent meta‐analysis of 97 studies confirmed a small but significant correlation between statistical learning and language abilities (Boeve, Zhou, & Bogaerts, 2024).

While most studies have investigated the statistical learning–language association in the typically developing population, results from populations with atypical language development can further inform our understanding of this relationship. Developmental disorders are often associated with greater heterogeneity in both language skills and other cognitive functions than typically developing individuals. This provides a richer context and a more robust basis for examining these associations. If deficits in statistical learning consistently co‐occurred with language difficulties in these populations, this would strengthen the case for the role of statistical learning as a fundamental mechanism underlying language acquisition and processing.

However, statistical learning is a multidimensional construct. Its measurement critically depends on task characteristics such as modality (auditory, visual), stimulus type (verbal, nonverbal), and the specific learning demands involved (e.g., segmentation, sequence learning, category learning; Bogaerts, Siegelman, & Frost, 2021; Lukics & Lukács, 2022). These modality‐ and stimulus‐specific constraints suggest that statistical learning may be better understood as a set of computations implemented across partly distinct neural systems than as a unitary mechanism (Bogaerts, Siegelman, Christiansen, & Frost, 2022; Conway, 2020; Frost et al., 2015).^1^ Therefore, interpreting individual differences requires careful specification of both task features and the computations they engage. In line with this, prior work suggests that associations between statistical learning and language can differ across tasks that rely on partly distinct components of learning. Studies in both typical and atypical development show that statistical learning tasks using verbal and auditory stimuli tend to exhibit stronger relations to language outcomes than nonverbal visual statistical learning tasks (Boeve et al., 2024).

The question of mapping between statistical learning tasks and learning constructs is particularly relevant in the context of language impairments, where differences in statistical learning have been attributed to a wide range of underlying mechanisms (Bogaerts et al., 2021). At the same time, studies increasingly show that atypical groups are highly heterogeneous, and that individual profiles of statistical learning and language ability can vary substantially within diagnostic categories (Arciuli & Conway, 2018). These considerations motivate the present study, which examines individual differences in statistical learning across children with developmental language disorder (DLD), attention deficit hyperactivity disorder (ADHD), autism spectrum disorder (ASD), and typical development (TD) using a single, consistently administered verbal auditory statistical learning paradigm. With this approach, our work joins ongoing efforts to provide more precise theoretical accounts of how statistical learning may contribute to language variation in developmental disorders.

DLD is a neurocognitive condition where language acquisition shows impairments at multiple levels in the absence of intellectual, sensory, or socioeconomic causes. Behavioral, cognitive, or sensorimotor symptoms are frequently present along with the primary impairment of language (Bishop, Snowling, Thompson, Greenhalgh, & the CATALISE‐2 consortium, 2017; Leonard, 2014). Individuals with DLD display great heterogeneity in the specific linguistic domain affected and the overall severity of the language difficulties and in impairments of other cognitive domains such as executive functioning (e.g., Henry, Messer, & Nash, 2012; Kapa & Erikson, 2019; Lancaster & Camarata, 2019; Leonard, 2014; Lukács, Ladányi, Fazekas, & Kemény, 2016; Marton, 2024) and procedural learning (Ullman & Pierpont, 2005). The vulnerability of statistical learning in the disorder has also been shown in several studies (Evans, Saffran, & Robe‐Torres, 2009; Haebig, Saffran, & Ellis Weismer, 2017; Hsu & Bishop, 2014; Kemény & Lukács, 2010; Lammertink, Boersma, Wijnen, & Rispens, 2020; Lukács & Kemény, 2014; Lukács, Lukics, & Dobó, 2021; Lum, Conti‐Ramsden, Morgan, & Ullman, 2014; Mayor‐Dubois, Zesiger, Van Der Linden, & Roulet‐Perez, 2014; Obeid, Brooks, Powers, Gillespie‐Lynch, & Lum, 2016).

ADHD is one of the most common developmental disorders with two leading clusters of symptoms, hyperactivity/impulsivity and inattention (American Psychiatric Association, 2013), both related to the impairment of executive functioning (Brocki & Bohlin, 2006; Thorell & Wåhlstedt, 2006). Individuals with ADHD are usually characterized by some form of mild language impairment as well (Bellani, Moretti, Perlini, & Brambilla, 2011). Most problems are observed at the pragmatic level of language (e.g., Geurts & Embrechts, 2008; Kim & Kaiser, 2000), and mixed results on the grammatical and lexical level indicate that there is no general language impairment in this disorder (Korrel, Mueller, Silk, Anderson, & Sciberras, 2017). Studies on statistical learning also show mixed results. Although most studies show intact statistical learning (e.g., Karatekin, White, & Bingham, 2009; Parks & Stevenson, 2018; Richards, Karvelis, Lawrie, & Seriès, 2020; Takács et al., 2017), multiple studies have found atypical learning patterns (e.g., Barnes, Howard, Howard, Kenealy, & Vaidya, 2010; Pedersen & Ohrmann, 2018; Rosas et al., 2010). However, some of these can be explained by impairments in executive functioning (Pedersen & Ohrmann, 2018).

ASD is a pervasive neurodevelopmental disorder characterized by a permanent impairment in socio‐communicative skills and by restricted and repetitive behavior and interests. Cognition is mainly affected in mentalization and executive functioning, but the disorder is also frequently associated with language deficits (American Psychiatric Association, 2013; Cantio, Jepsen, Madsen, Bilenberg, & White, 2016). Although pragmatic skills are affected in almost all cases, and syntactic and lexical abilities can also show atypical patterns, there is a wide variation in language abilities across individuals with ASD from non‐verbal individuals to above‐average language performance. Mixed results from previous studies on statistical learning indicate that there is a high variability in this ability as well: Many studies argue for intact statistical learning abilities (Foti, De Crescenzo, Vivanti, Menghini, & Vicari, 2015; Haebig et al., 2017; Obeid et al., 2016; Pesthy et al., 2023), while others suggest impaired (e.g., Mostofsky, Goldberg, Landa, & Denckla, 2000; Parks, Griffith, Armstrong, & Stevenson, 2020), or the opposite, enhanced statistical learning (Roser, Aslin, McKenzie, Zahra, & Fiser, 2015).

These results of pronounced impairments in statistical learning in individuals with DLD and more mixed results in those with ADHD and ASD align with findings from TD and emphasize the role of statistical learning in shaping language ability. However, they provide only indirect evidence for the relationship between statistical learning and language. Since both statistical learning deficits and language impairments are observed in ADHD, ASD, and DLD (although to different degrees and in various patterns), one could argue that weaknesses in statistical learning contribute to language difficulties in these disorders. Yet, only a few studies have examined this relationship directly in developmental disorders. Evans et al. (2009) showed that statistical learning as measured by a speech segmentation task correlated with vocabulary size in both TD and DLD. Hsu and Bishop (2014) showed indirect evidence for the statistical learning–language relationship in DLD: In a serial reaction time task, DLD groups showed impaired performance, compared to an age‐matched TD group, but had comparable performance to a TD group matched by grammatical ability. In the case of ASD, Parks et al. (2020) found an association between visual segmentation and receptive language abilities in individuals with ASD. Haebig et al. (2017) found that, in contrast with children with TD and DLD, the statistical learning abilities of children with ASD measured with a speech segmentation task correlated with their receptive and expressive vocabulary and grammatical skills. Hu, Kozloff, Owen Van Horne, Chugani, and Qi (2024) assessed the statistical learning abilities of children with ASD with segmentation tasks in the linguistic and nonlinguistic domains and in the visual and auditory modalities. They found that the composite performance in linguistic segmentation tasks correlated with children's linguistic abilities, while they found no such correlation with nonlinguistic segmentation tasks.

In addition to the scarcity and mixed findings of studies in DLD and ASD, to our knowledge, no studies have investigated the relationship between statistical learning and language abilities in ADHD. Also, the relationship of statistical learning with language was assessed predominantly by using a speech segmentation task. Therefore, it remains unclear whether deficits in statistical learning directly contribute to language impairments in developmental disorders, and this hypothesis requires further testing across a wider range of populations and with more diverse statistical learning tasks targeting multiple domains, modalities, and statistical regularity types.

Developmental disorders are increasingly viewed as arising from continuous variation in underlying cognitive and affective traits (Astle, Holmes, Kievit, & Gathercole, 2022; Michelini et al., 2024). In this view, neurodevelopmental conditions do not represent discrete categories, but rather reflect specific constellations of trait values within a multidimensional space, and a given condition can be understood as the outcome of an individual's position along these trait dimensions. As the traits that contribute to these conditions are continuously distributed in the population, neurodevelopmental conditions form a continuum with TD. This continuum‐based dimensional view is generally considered for ASD (Abu‐Akel, Allison, Baron‐Cohen, & Heinke, 2019; Bölte, Westerwald, Holtmann, Freitag, & Poustka, 2011; Robinson et al., 2011) and, to some extent, ADHD (Fair, Bathula, Nikolas, & Nigg, 2012) and has also been proposed for DLD (Lancaster & Camarata, 2019). The dimensional view implies that individuals without a formal diagnosis can have cognitive profiles similar to those with a diagnosis, although with milder impairments or patterns of strengths and weaknesses more similar to those in TD. If language is built upon general cognitive abilities, then language impairment in these disorders is likely not a primary deficit but rather a consequence of broader cognitive weaknesses across different neurodevelopmental conditions. Based on these assumptions, language abilities of children with developmental disorders and those with TD also lie on a multidimensional continuum, shaped by differences in underlying cognitive factors. This perspective has the potential to provide a better understanding of how cognitive abilities, for instance, statistical learning, shape language in both typical and atypical development.

In the present study, we took a dimensional perspective to explore the relationship between statistical learning and language abilities in a sample of children with TD along with children with DLD, ADHD, and ASD. By examining TD children alongside those with DLD, ADHD, and ASD, we extend this dimensional view to an empirical design that goes beyond the traditional focus on single diagnostic groups. Considering these profiles together, each characterized by distinct but partly overlapping patterns of cognitive and language functioning, allows us to test whether domain‐general mechanisms such as statistical learning, short‐term and working memory, and perceptual speed predict language outcomes irrespective of diagnostic category. This approach enables us to identify shared and condition‐specific cognitive influences on language, which provides theoretical grounds for comparing multiple neurodevelopmental groups within a single framework. Our aim was to investigate whether variability in statistical learning ability could explain differences in vocabulary size and grammatical abilities in this neurodivergent population.

We use an auditory verbal artificial grammar learning (AGL) task for assessing statistical learning. Unlike widely used segmentation paradigms, where learning relies on adjacent dependencies (i.e., differences in transitional probabilities), AGL paradigms assess sensitivity to higher‐order statistical dependencies in addition to adjacent dependencies. For example, a transition such as *XY* may be legal only if it is followed by *Z*. This allows us to examine sensitivity to more complex sequential regularities than those typically captured by segmentation paradigms, of the kind that may also be at work in language acquisition and use.

However, statistical learning and language seem to share cognitive resources. For example, individual differences in attention (Turk‐Browne, Jungé, & Scholl, 2005), short‐term and working memory (Hendricks, Conway, & Kellogg, 2013; Kidd et al., 2023; Misyak & Christiansen, 2012), and cognitive control processes (Horváth, Török, Pesthy, Nemeth, & Janacsek, 2020; Pedraza et al., 2024) influence statistical learning outcomes (for comprehensive studies, see Kidd et al., 2023; Lukács, Ugrin, & Lukics, 2026), and these domain‐general cognitive abilities have a role in language acquisition and use as well. Therefore, it is necessary to control for their effects when investigating the direct relationship between statistical learning and language. This consideration is particularly important with the targeted atypical groups, as DLD, ADHD, and ASD are characterized by impairments in short‐term and working memory, cognitive control, and perceptual processing in one or more modalities (Mayes & Calhoun, 2007; Oliveras‐Rentas, Kenworthy, Roberson, Martin, & Wallace, 2012; Zapparrata, Brooks, & Ober, 2023). To control for these confounding effects, we also assessed individual differences in perceptual speed and short‐term and working memory. We had the following research questions:

1. Is statistical learning ability a relevant predictor of vocabulary size and grammatical abilities in this neurodivergent sample?
2. Does statistical learning remain a relevant predictor of vocabulary size and grammatical abilities when individual differences in perceptual speed and working memory are taken into account?
3. Is this relationship between cognitive abilities and language different across neurodevelopmental groups (TD, ASD, DLD, ADHD)?

## 2 Method

Task stimuli and data are provided in the Supporting Information, available here: https://doi.org/10.17605/OSF.IO/3V7NA.

### 2.1 Participants

A total of 102 native Hungarian‐speaking children took part in this study, 20 with DLD (five girls and 15 boys), 26 with ASD (four girls and 22 boys), 24 with ADHD (six girls and 18 boys), and 32 TD (10 girls and 22 boys). Children with DLD were recruited from two special schools for children with language and speech difficulties in Budapest. They had a formal ICD‐10 (International Classification of Diseases, Tenth Revision) (World Health Organization, 1992) based diagnosis of Expressive language disorder (F80.1), Mixed receptive‐expressive language disorder (F80.2) or Developmental disorder of speech and language, unspecified (F80.9) by the Expert and Rehabilitation Committee on Cognitive Capacities of the local Pedagogical Professional Services. Participants of the ADHD group were recruited from the Division of Neurodevelopmental Disorders of a children's hospital in Budapest. Participants of the ASD group were recruited from the same Budapest hospital division and by a special education teacher specialized in ASD in a mid‐sized city in northeastern Hungary. Each child had a previous, DSM‐5 (Diagnostic and Statistical Manual of Mental Disorders, Fifth Edition) (American Psychiatric Association, 2013) based diagnosis of ADHD or ASD, which was established by a child psychiatrist. The ASD diagnoses were made by senior psychiatrists specializing in child and adolescent psychiatry. The diagnosis was based on a detailed history and observational data both from home and kindergarten or school, using the DSM‐5 diagnostic criteria. ADHD diagnoses were also made by a senior psychiatrist specializing in children and adolescent psychiatry, based on a detailed history and observational data both from home and school, using DSM‐5 diagnostic criteria. The parent and teacher versions of the Swanson, Nolan, and Pelham, version IV (SNAP‐IV) questionnaire (Swanson et al., 2001) was used to gather information about home and school behavior. We used subscale cutoff scores suggested by the authors (Bussing et al., 2008): (Parent Inattention subscale cutoff score: 2.4, Parent Hyperactivity/Impulsivity subscale cutoff score: 1.8, Teacher Inattention subscale cutoff score: 1.8, Teacher Hyperactivity/Impulsivity subscale cutoff score: 1.8.) Most of the children in the ADHD group were treated with medication (19 children with methylphenidate, three children with other medication). Based on the recommendations of the hospital's psychiatrist, if children were treated with methylphenidate, we asked the parents to skip the dose in the 8‐h interval preceding testing. Children with documented comorbid developmental disorders were excluded from all diagnostic groups. Only participants with a single, confirmed primary diagnosis were included. Although underdiagnosing neurodevelopmental conditions is a recognized challenge in Hungary, all available clinical records and assessments were reviewed to minimize the inclusion of children with undetected comorbidities. The TD participants were recruited from three schools in Budapest, one school in a suburban town near Budapest, and one school in a village in southeastern Hungary. None of the TD participants had a previous diagnosis of any psychiatric, neurological, or developmental disorder.

We matched groups on age and nonverbal IQ, assessed by the Raven's Colored Progressive Matrices (Raven, Court, & Raven, 1990). Participants with an IQ score below 85 were not included in the study. Descriptive statistics of the demographic data and task performances are presented in Table 1. No specific demographic data about socioeconomic status were collected.

**Table 1:** Demographic data and descriptive statistics of task performances in the typical development (TD), attention deficit hyperactivity disorder (ADHD), autism spectrum disorder (ASD), and developmental language disorder (DLD) groups

Variable	TD	ADHD	ASD	DLD
age (years)	11.8 (2.1)	12.0 (1.2)	11.4 (1.8)	12.1 (1.8)
boys/girls	22/10	18/6	22/4	15/5
IQ	104.5 (12.3)	107.5 (15.3)	108.8 (10.6)	105.2 (15.2)
language: expressive vocabulary	0.91 (0.04)	0.88 (0.07)	**0.78 (0.15)**	0.87 (0.06)
language: sentence repetition	0.71 (0.12)	0.62 (0.16)	**0.53 (0.25)**	**0.46 (0.11)**
statistical learning: AGL response time	0.06 (0.10)	0.02 (0.08)	0.02 (0.11)	0.05 (0.13)
statistical learning: AGL offline	0.49 (0.08)	0.47 (0.07)	0.45 (0.12)	0.45 (0.08)
perceptual speed: visual response time	0.37 (0.08)	0.40 (0.11)	0.41 (0.14)	0.42 (0.11)
perceptual speed: auditory response time	0.34 (0.07)	0.36 (0.10)	0.39 (0.11)	0.37 (0.08)
working memory: forward digit span	5.41 (0.91)	4.91 (1.06)	**4.79 (1.47)**	**4.33 (0.77)**
working memory: backward digit span	4.14 (1.36)	**3.36 (0.90)**	**3.50 (1.18)**	**3.22 (1.00)**
working memory: n‐back	20.9 (20.0)	25.1 (18.6)	12.8 (18.3)	16.9 (22.6)

*Note*. Means are displayed with standard deviations in parentheses. For demographic data and descriptive statistics, group values that were significantly different from values of the TD group are in bold (see the Appendix in the Supporting Information for the analyses).

Abbreviation: AGL, artificial grammar learning.

### 2.2 Tasks

In this study, we focused on expressive vocabulary and sentence repetition as language outcome measures for two reasons. First, these two subtests from the Complex Test of Spoken Language Skills (KOBAK) test battery (Lukács & Kas, 2024) provide brief but psychometrically robust indices of core expressive language domains, namely, lexical access and morphosyntactic processing. Second, they are both sensitive indices of language weaknesses in neurodevelopmental conditions, making them suitable indicators for examining the cognitive predictors of language performance.

#### 2.2.1 Expressive vocabulary

To estimate participants’ vocabulary size, we used two naming subtests of the KOBAK test battery to calculate an *expressive vocabulary* index. Participants were presented with individual pictures, and they were instructed to name what the pictures depicted. In the *Naming: Nouns* subtest, children had to name objects, and in the *Naming: Verbs* subtest, they had to name actions presented in the pictures. Both subtests started with a practice session of two trials, which was followed by a test session of 60 trials. Different trials varied in length and frequency of the target word. Multiple correct answers were accepted for each trial, and the evaluation was carried out by using a list based on the responses of a larger sample. Accuracy rates were calculated by dividing the sum of the correct responses in the two subtests by the total number of trials, yielding the *language: expressive vocabulary* index.

#### 2.2.2 Sentence repetition

Participants’ grammatical performance was assessed with the *Sentence Repetition* subtest of the KOBAK test battery. This task started with two practice trials followed by 50 test trials. In each trial, the experimenter read an individual sentence, and participants were instructed to repeat it exactly as they heard it. Although sentence repetition involves verbal memory, performance is known to reflect the interaction of memory capacity and grammatical representations rather than simple serial recall. Accuracy systematically varies with syntactic complexity, and the task is widely used as a clinical marker of morphosyntactic impairment (Conti‐Ramsden, Botting, & Faragher, 2001; Klem et al., 2015; Riches, 2012).

Sentences were presented in incremental order of length, and participants were tested with two sentences in each length: (1) with a simple sentence and (2) with a complex sentence. Sentence length varied between 6 and 30 syllables, and items were presented in increasing length and complexity. Complex items included relative clauses, subordination, and multiple embeddings. Thus, items varied not only in length but also in morphosyntactic complexity. The correct repetition of the sentence was scored with three points, two points were given in the case of one error, one point in the case of two errors, and zero points if there were three or more errors in the repetition. Errors were scored at the level of individual morphemes, including content and function words, elements of compounds, and affixes. An error was counted when a morpheme was omitted, added, substituted, or recalled in the wrong position in the repeated sentence. The task was finished after three consecutive zero scores or after completing the 50 test trials (whichever happened first). The *language: sentence repetition* index was calculated by dividing the sum of scores by three times the number of trials for each participant.

#### 2.2.3 AGL

**Fig. 1:**
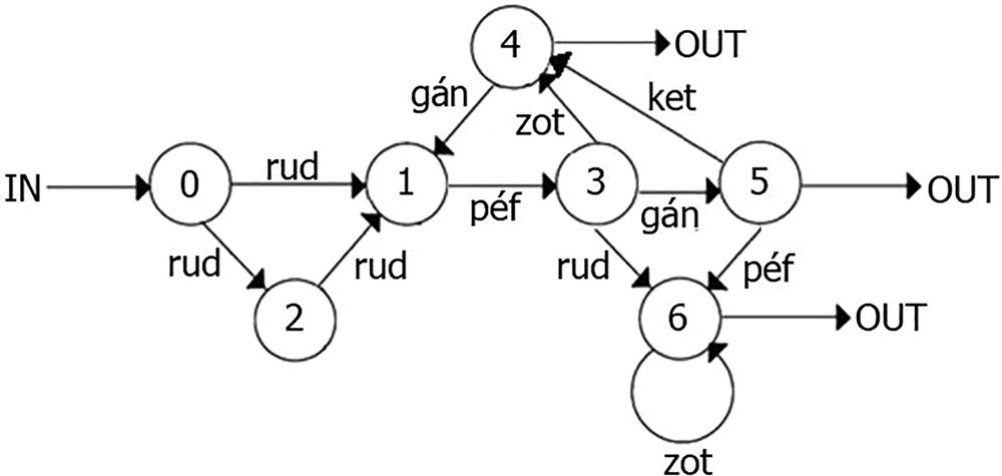
The miniature grammar (based on Knowlton & Squire, 1996).

We assessed statistical learning using a modified version of the AGL task by Knowlton and Squire (1996). Participants listened to sentences generated from a miniature grammar (see Fig. 1) and lexicon consisting of five consonant‐vowel‐consonant nonwords (péf /peːf/, rud /rud/, gán /ɡaːn/, ket /kɛt/, zot /zot/). Words had a uniform pitch of 125 Hz and a duration of 400 ms. They were presented with an interstimulus interval (ISI) of 50 ms within sentences and 100 ms between sentences.

Based on the grammar, 34 three‐ to seven‐word grammatical sentences were generated. From these, 26 sentences were used during the online training phase, and eight sentences were used during the offline tests. The experiment began with an online training phase implemented as a target detection task. Participants were instructed to press the spacebar when they heard the predefined target word (péf /peːf/), and to press the “A” key for all other words.

The training phase consisted of five blocks. Blocks TRN1–TRN3 were structured training blocks containing the same 26 grammatical sentences. Block RND4 was an unstructured random block consisting of 26 sentences where words were arranged in a random order. Block REC5 was a structured recovery block identical to the training blocks. Sentences in all blocks were presented in a randomized order. Each training block contained 42 target occurrences, while the random block contained 43. Reaction times and accuracy were recorded for all target responses across blocks. As targets become predictable based on the grammar, we expected learning to be reflected by increasing accuracy and decreasing reaction times across the structured training blocks (TRN1–TRN3), a disruption of performance in the random block (RND4), and a recovery of performance in the final structured block (REC5). From this online phase, the *statistical learning: AGL response time* index was calculated by averaging the differences between the random and the neighboring structured blocks: ((median RT_RND4_ ‐ median RT_TRN3_) + (median RT_RND4_ ‐ median RT_REC5_)) / 2. Exclusion criteria are detailed in the Appendix in the Supporting Information.

Following the online phase, participants were assessed using two offline tasks. First, they completed a two‐alternative forced choice (2AFC) task in which they had to select the string that was more similar to the previously presented language from a grammatical and an ungrammatical string. This task consisted of two blocks: one testing sensitivity to the grammar in complete sentences (16 trials) and one in sentence fragments (bigrams and trigrams; 22 trials). Each of the eight grammatical complete sentences was a novel sentence that was not used in the training phase. They were presented in two 2AFC trials. Grammatical sentence fragments were present in sentences of the training phase and were also presented in two 2AFC trials. Accuracy was calculated separately for the two blocks and averaged to yield the *AGL 2AFC* index. Finally, participants completed a production task in which they were instructed to complete incomplete grammatical sequences by selecting one of three possible words. The task included 24 trials involving grammatical trigrams and complete sentences. The position of the missing item varied across trials. Accuracy in this task yielded the *AGL production* index. For a comprehensive presentation of the 2AFC task and production task trials and stimuli, see the Supporting Information. The *statistical learning: AGL offline* index was calculated by averaging the *AGL 2AFC* and *AGL production* indices.

#### 2.2.4 Perceptual speed task

To test perceptual speed, we used a reaction time task that measured the speed of stimulus processing in the auditory and visual modalities. In the first, visual block, children were instructed to look at a blank screen and focus on the appearance of a drawing of a ball in the middle of the screen and to press the spacebar as fast as they could each time when the ball appeared. The visual block was followed by an auditory block, in which participants had to focus on a tone presented via headphones and to press the spacebar when the tone was presented. For both modalities, 32 trials were presented with ISIs varying randomly between 1223 and 4988 ms for the visual modality and 1041 and 4883 ms for the auditory modality. We calculated perceptual speed indices from median RTs for both modalities: *perceptual speed: visual response time* and *perceptual speed: auditory response time*.

#### 2.2.5 Digit span task

We assessed short‐term and working memory capacity by online versions of the digit span tasks (Racsmány, Lukács, Németh, & Pléh, 2005). In two subtasks, sequences of spoken digits were presented, and participants were instructed to type them in the same (forward digit span) or in the reversed (backward digit span) order. Tasks started with the presentation of three‐digit‐long sequences, and the length of sequences increased as the task proceeded, with a maximum length of 10 digits. Participants were presented with four trials within each length, and the task was terminated after three incorrect responses within the same length. For each task, the longest sequence with at least two correct responses was taken as the measure: *working memory: forward digit span* (measuring short‐term memory) and *working memory: backward digit span* (indicating working memory).

#### 2.2.6 N‐back task

We used an n‐back task to measure working memory and updating capacity. In this task, letters were presented individually in the middle of the screen, and children were instructed to press the spacebar for every item that was identical to the one presented two steps earlier and button “A” for every other trial. The task consisted of four blocks altogether: a 1‐back, two 2‐back, and one 3‐back block. Each block consisted of 60 trials (10 targets, 10 lures, and 40 foils). Blocks started with a fixation cross for 1000 ms, followed by individual trials presented for 500 ms with an ISI of 1500 ms. As in the case of children, only 2‐back blocks measured performance reliably (1‐back being too easy and 3‐back being too difficult), we used only these blocks in our analyses. We calculated the *working memory: n‐back* score for each participant by subtracting the rate of false alarms from the rate of hits from the two‐back blocks (((hits/10) − (false alarms/50)) * 100).

#### 2.2.7 Index reliabilities

In the full standardization sample of 495 children aged 3–13 years, Cronbach's α was 0.98 for *language: expressive vocabulary* and 0.97 for *language: sentence repetition* (Lukács & Kas, 2024). For the indices from the cognitive predictor tasks, we estimated split‐half reliability on item‐level data from the present sample. In each of 1000 iterations, trials were randomly split into two halves within each participant. The index of interest was then recomputed within each half exactly as in the main analyses. We then calculated correlations between the two halves using Spearman–Brown correction. The digit span indices were exceptions regarding the split‐half calculation methods: Although item‐level accuracy is available, the index is a span score from adaptive testing because the task terminates at the first length at which the task continuation criterion (three incorrect trials at a given sequence length) fails. Therefore, the halves of such an administration are not parallel: three (75%) incorrect responses by sequence length cannot be mapped to the two halves, as the number of trials per sequence length is two. For this reason, as an approximation, we computed split‐half reliability for a total‐correct score (see the Supporting Information). Total‐correct scores show a 0.85 and 0.93 correlation with span scores for the forward and backward subtasks, respectively. The results of this approximation are in line with data from the literature indicating good reliability: The English version of the task has a test–retest reliability of 0.76 in a clinical child sample (Watkins et al., 2022).

We report the medians and the 95% confidence intervals (CI) for task indices in Table 2.

**Table 2:** Split‐half reliabilities for predictor indices

Index	*r* Median	*r* 95% CI
statistical learning: AGL response time	0.35	[0.03, 0.57]
statistical learning: AGL offline	0.51	[0.35, 0.64]
perceptual speed: visual response time	0.92	[0.84, 0.96]
perceptual speed: auditory response time	0.99	[0.90, 1.00]
working memory: forward digit span	0.91	[0.88, 0.93]
working memory: backward digit span	0.94	[0.92, 0.96]
working memory: n‐back	0.71	[0.60, 0.80]

*Note*. Split‐half reliabilities could not be performed on *working memory*: *forward digit span* and *working memory*: *backward digit span*; therefore, they were calculated for total‐correct scores for these indices.

Abbreviation: AGL, artificial grammar learning.

### 2.3 Procedure

The tasks described above were part of a larger test battery. As a first step, all children were individually tested with the Raven's Colored Progressive Matrices test, as the criterion of inclusion in further testing was an IQ score at or above 85. Language tasks were also administered individually, while general cognitive tasks were administered individually in the atypical (DLD, ASD, and ADHD) groups and in small groups of two to four in the TD group. Children with DLD and TD were tested in a silent room of the school, after teaching hours, while children in the ADHD and ASD groups were tested after school, in a silent room of the hospital where they were diagnosed. Children completed the tasks in 45–90 min sessions (depending on the time available and the child's limits of attention), 1–4 weeks apart. The Raven's test and the language tasks were presented on paper, and all other tasks were coded in PsychoPy (Peirce et al., 2019) and data were collected on computers via the Pavlovia online platform. Acoustic stimuli were presented over headphones (SONY MDR‐ZX310R).

Participants were tested with the informed consent of their parents, in accordance with the principles set out in the Declaration of Helsinki. The study was approved by the United Ethical Review Committee for Research in Psychology (Approval Number: EPKEB‐2018/87).

### 2.4 Data analysis

Our outcome variables were *language*: *expressive* *vocabulary* and *language: sentence repetition*. Our predictor variables were children's results in the other tasks outlined above. We provide two sets of analyses. First, we looked at individual predictors and how these correlate with each other and the outcome variables, using bootstrapped correlations. Second, we provide machine learning models to capture broad relationships between our two outcome variables and the predictor variables.

The dataset has missing values (we only have complete observations for 71/102 children, see the Appendix in the Supporting Information), and the predictor variables are correlated with one another. This includes the neurodevelopmental groups, meaning that testing interactions of *neurodevelopmental group* and the other predictor variables in a linear model framework results in collinearity and uninterpretable models.

Data with correlated variables, missing observations, and a large predictor/observation ratio are particularly challenging to model. We used a machine learning algorithm called XGBoost (“Extreme Gradient Boosting”) to model participant responses in the sentence repetition and the expressive vocabulary tasks. XGBoost combines many small, simple decision tree models in sequence to build a large model. Each new tree tries to correct the errors made by the previous ones in the sequence, resulting in a more accurate final prediction. XGBoost naturally handles missing values and can manage many correlated variables. It is broadly accurate on high‐dimensional tabular data and can be robust to overfitting (Chen & Guestrin, 2016). For our purposes, its main value lies in being able to deal with incomplete cases naturally. Not all participants have data from all tests, but since the method fits small models on incomplete subsets, it substantially mitigates this problem.

We fitted two boosting models, predicting (1) *language: expressive vocabulary* score and (2) *language: sentence repetition* score from *neurodevelopmental group*, *statistical learning: AGL* *response* *time*, *statistical learning: AGL offline*, *perceptual speed: visual* *response time*, *perceptual speed: auditory response time*, *working memory: forward digit span*, *working memory: backward digit span*, and *working memory: n‐back* scores (see Section 2.2 for the description of task indices). We selected hyperparameters via grid search over tree depth, learning rate, subsampling, and regularization parameters (see Appendix Table A1 in the Supporting Information). We selected the best model using 10‐fold cross‐validation and mean squared error. The best model had an MSE of 0.008 for expressive vocabulary and 0.002 for sentence repetition. This translates to a root mean squared error of 0.09 and 0.05, respectively. This means that, during cross‐validation, model predictions deviated from observed values by about 9% for expressive vocabulary and 5% for sentence repetition. We report the best models below.

The XGBoost model was fitted using xgboost (Chen & Guestrin, 2016) and sklearn (Pedregosa et al., 2011) in Python, and figures were created using ggplot2 (Wickham, 2016) in R.

## 3 Results

**Fig. 2:**
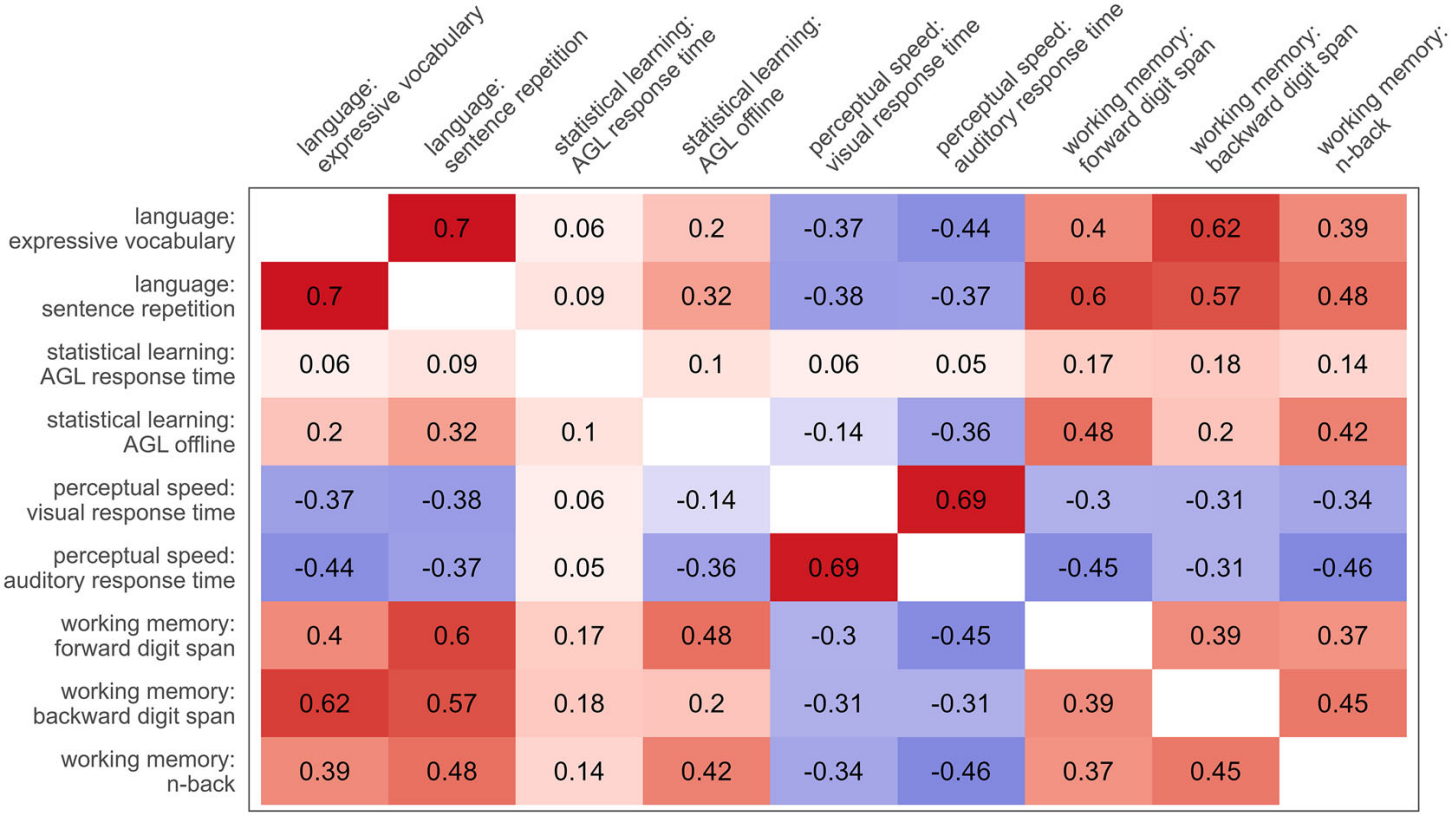
Spearman correlations between cognitive predictor and language outcome variables.

The Spearman correlations among the predictor and outcome variables can be seen in Fig. 2. Closely related measures are highly correlated, which is less surprising (e.g., *perceptual speed: auditory* and *visual response time* have a Spearman's rho of 0.69). However, all predictors show mild to moderate correlations with one another. This is what makes modeling this dataset particularly challenging.

**Fig. 3:**
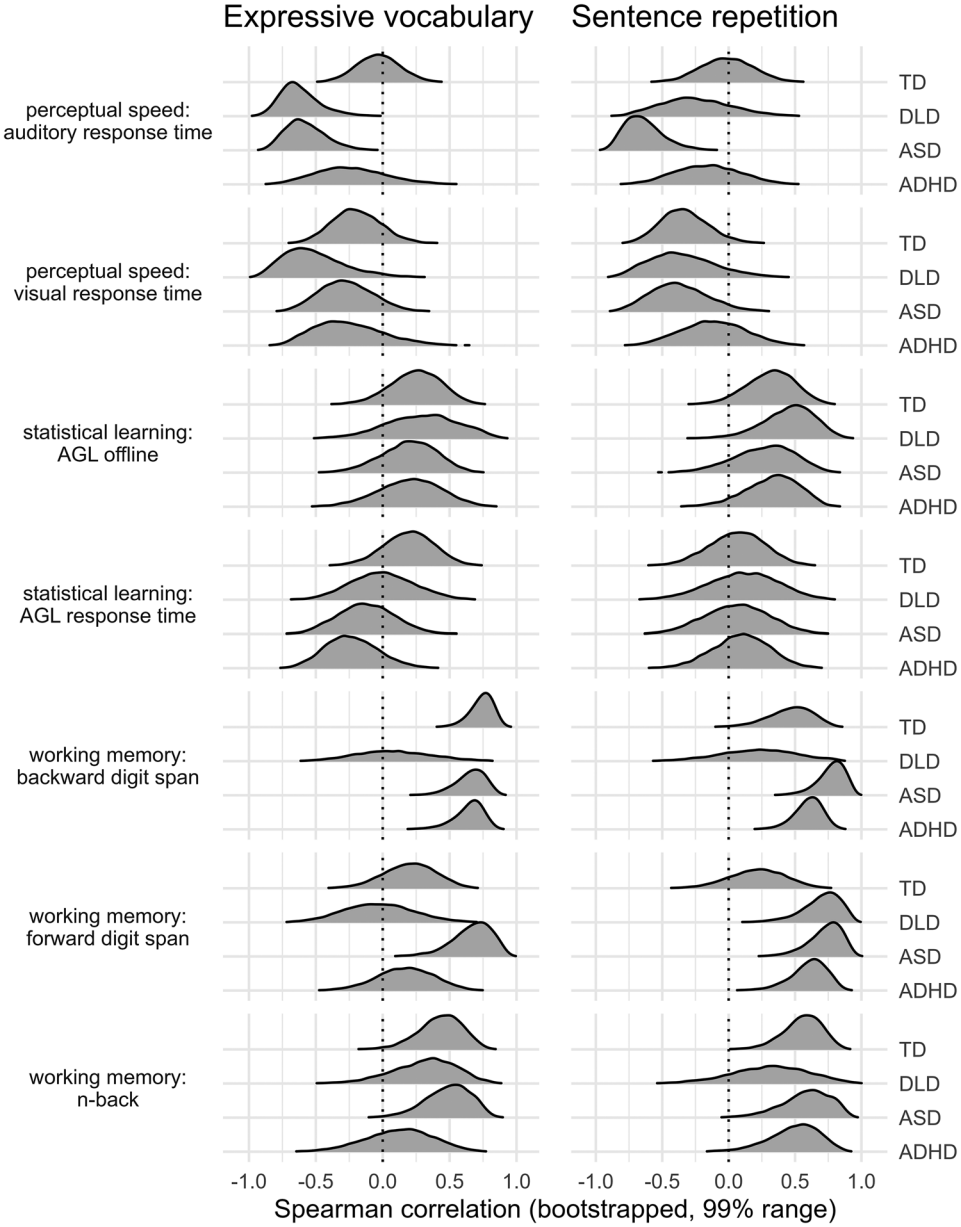
Bootstrapped correlations for expressive vocabulary, sentence repetition, and the cognitive predictor variables across the neurodevelopmental groups. *Note*. The density plots show 99% of all bootstrapped Spearman correlation coefficients between the language outcome measures (*language: expressive vocabulary* and *language: sentence repetition*) and the cognitive predictor variables within each diagnostic group, based on individual participants’ scores. We bootstrapped Spearman correlation coefficients using 10,000 iterations to account for the uncertainty of the correlations. In each iteration, we took a random sample from the original data and calculated Spearman's rho and then visualized the distribution of 99% of the bootstrapped observations to give a sense of the most likely relationship between variables. We use 99%, rather than the more conventional 95%, to emphasize how repeated independent comparisons can inflate correlations. (We might find a major difference in how a given group performs relative to other groups in expressive vocabulary or sentence repetition across one of the predictor measures, but this will be one of 56 parallel analyses.) ADHD, attention deficit hyperactivity disorder; ASD, autism spectrum disorder; DLD, developmental language disorder; TD, typically developing.

Estimating interactions between *neurodevelopmental group* and the cognitive predictors was not feasible given the group sizes (20–32) and the degree of collinearity among predictors. We instead examine whether predictor–outcome relationships differ across groups using bootstrapped correlations stratified by diagnostic category. The bootstrapped Spearman correlations of the seven cognitive predictor variables and the two language ability outcome variables, across the four groups, can be seen in Fig. 3. The correlation distributions show that most of the neurodevelopmental groups pattern together across all outcome variables, with the possible exceptions of the *perceptual speed: auditory response time* and *working memory: digit span* indices, where ASD and DLD groups have slightly different, if highly variable, patterns of cognitive–language relationships. Overall, short‐term and working memory indices, especially the digit span indices, are the most predictive of expressive vocabulary and sentence repetition. From the statistical learning indices, the offline measure shows a stronger correlation with the outcome variables. The effect of the predictors on expressive vocabulary and sentence repetition is remarkably similar.

**Fig. 4:**
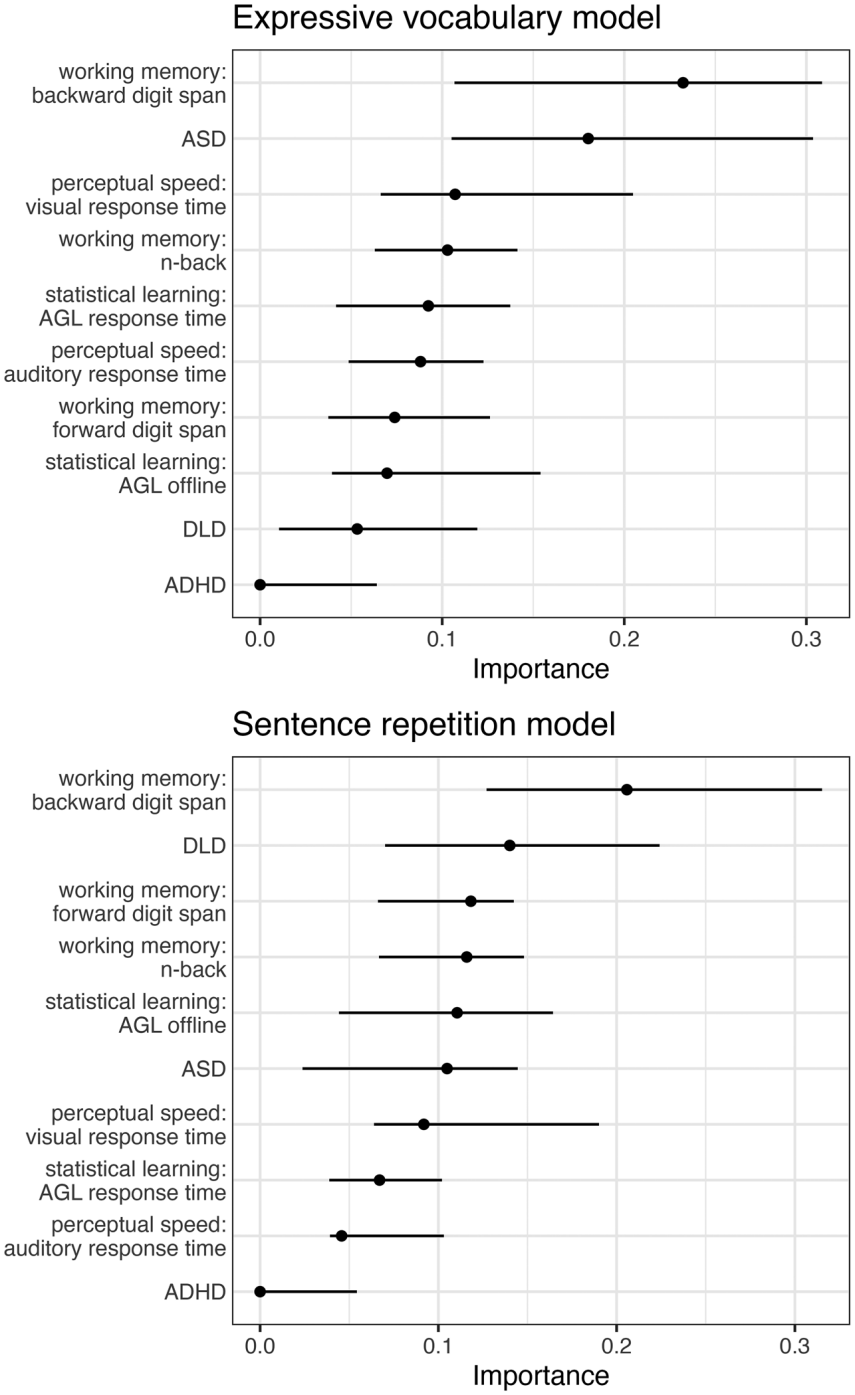
Scaled variable importances from the models predicting *language: expressive vocabulary* (top) and *language: sentence repetition* (bottom) Note: Whiskers show 95% confidence intervals for variable importances.

The gradient boosting models allow us to quantify these relationships while accounting for the effects of all predictors in a single model. Variable importances from the best models on *language: expressive vocabulary* and *language: sentence repetition* can be seen in Fig. 4. We estimated 95% bootstrap confidence intervals over 1000 resamples and report the proportion of resamples in which each feature received zero importance as a conservative *p*‐value analog. Relative importance expresses how much variation is explained by a particular predictor across many small models. In decision trees, variable importance reflects how much each variable helps to explain the variation in the outcome, often capturing both its individual effect and its interactions with other variables (since, put simply, the effect of variables can depend on the effect of previous variables higher up in the tree). As all predictors were entered simultaneously, variable importance scores index the contribution of each predictor to explaining variation in the language outcomes, over and above the contribution of other predictors in the model, rather than simple bivariate associations.

In the case of both *language: expressive vocabulary* and *language: sentence repetition*, *working memory: backward digit span* was the most important predictor. In the case of *expressive vocabulary*, ASD was an important predictor, and in the case of *sentence repetition*, DLD was a less important, but somewhat notable, predictor. These effects of *neurodevelopmental group* reflect group differences in language outcomes: The mean performance of the ASD group was lower than the TD group in *expressive vocabulary*, and all neurodivergent groups (ADHD, ASD, and DLD) had lower performance than the TD group in *sentence repetition*; this difference was the largest in the case of the DLD group.

## 4 Discussion

The aim of our study was to explore the relationship between statistical learning and language abilities in children with TD and those with neurodevelopmental conditions, including DLD, ADHD, and ASD. Our approach was grounded in a dimensional view of development, in which cognitive and language abilities are considered to vary continuously across individuals with and without formal diagnoses. We specifically investigated whether individual differences in statistical learning are related to variability in vocabulary and grammatical performance and whether this relationship holds when accounting for the influence of domain‐general cognitive abilities, including perceptual speed and working memory. Our study extends previous work by examining the relationship between statistical learning and language abilities across multiple neurodevelopmental conditions within a single experimental design. By including children with DLD, ASD, ADHD, and TD, it takes a broad developmental perspective that goes beyond the traditional focus on either single diagnostic groups or typically developing populations. We also investigated whether this relationship persists if other cognitive abilities known to affect language development are taken into account. This inclusive approach supports more generalizable insights into the role of statistical learning in language acquisition and impairment and helps identify both shared and condition‐specific cognitive mechanisms across developmental profiles. We found that on the level of the overall group, individual differences in statistical learning (*AGL offline*) positively correlated with both vocabulary and grammatical performance. However, when individual differences in perceptual speed and working memory capacity were taken into account, verbal working memory, as measured by *backward digit span*, emerged as the most influential predictor. Bootstrapped correlation analyses across the neurodevelopmental groups showed no substantial difference in the relationship between cognitive and language abilities.

An important consideration when interpreting the relative contribution of the cognitive predictors concerns differences in measurement reliability. In the present data, the predictors varied substantially in this respect: the split‐half reliability of the *statistical learning: AGL offline* measure was only 0.51, a level substantially lower than the estimated reliability of the strongest predictor, *working memory: backward digit span* (0.94 derived from the approximation calculations). Because lower reliability reflects a larger proportion of measurement noise, correlations involving less reliable variables are necessarily attenuated. Consequently, the observed differences in relative importance between statistical learning and the other predictors may, in this case, particularly reflect differences in measurement reliability. In particular, the comparatively high importance of the *working memory: backward digit span* index may partly reflect its higher reliability. Likewise, the low reliability of *statistical learning: AGL response time*, which was only 0.35, likely contributed to the low correlation between the index and the language outcomes.

Our reliability estimates are in line with the literature: Statistical learning tasks, especially those using linguistic stimuli, often have low internal consistency in both child and adult samples (Arnon, 2020; Kidd et al., 2023; Pavlidou & Bogaerts, 2019; Siegelman, Bogaerts, Elazar, Arciuli, & Frost, 2018; but see serial recall indices in Kidd et al., 2023; Stärk, Kidd, & Frost, 2022, for examples of good internal consistency). Poor task reliability makes null or weak associations difficult to interpret since attenuated correlations may be the result of measurement noise rather than the absence of a relationship between statistical learning and language (e.g., Conway, Arciuli, Lum, & Ullman, 2019). The associations we observed for the statistical learning indices should therefore be read as likely underestimates.

The positive correlations between statistical learning and language are consistent with previous findings in the typically developing population (e.g., Kidd, 2012; Kidd & Arciuli, 2016; Kidd et al., 2023; Misyak et al., 2010; Misyak & Christiansen, 2012; Spencer et al., 2015; Torkildsen et al., 2019; Yu & Smith, 2007). The present results extend this association to a broader sample including children with neurodevelopmental conditions, supporting the view that sensitivity to statistical regularities may contribute to language acquisition across a range of developmental profiles. When perceptual speed and working memory were included in the models, descriptively, statistical learning accounted for a smaller proportion of the variance in *expressive vocabulary* and *sentence repetition* than verbal working memory measured by *backward digit span*. For both language outcomes, *working memory: backward digit span* emerged as the most influential predictor.

The results regarding the effect of verbal working memory are also supported by a substantial body of previous research, pointing out its role in shaping language abilities. Verbal working memory has been identified as a strong predictor of individual differences in language development, including vocabulary growth, syntactic processing, and sentence production (Adams & Gathercole, 2000; Alloway, 2009; Baddeley, Gathercole, & Papagno, 1998; Montgomery, Magimairaj, & Finney, 2010). This association has been documented both in typically developing children and in those with neurodevelopmental conditions. For instance, children with DLD often show reduced verbal working memory capacity, which is closely related to their grammatical difficulties (Archibald & Gathercole, 2007). Similar effects have been reported in children with ASD and ADHD, where working memory limitations are linked to language comprehension and expression difficulties (Joseph, McGrath, & Tager‐Flusberg, 2005; Kofler et al., 2011). In the present study, verbal working memory was consistently associated with both vocabulary and grammatical performance, suggesting that the ability to temporarily maintain and manipulate verbal material is a central factor in language performance across different developmental profiles.

While verbal working memory had a stronger effect than statistical learning on language outcomes in the analyses, this does not necessarily imply that statistical learning does not have an important role in shaping language abilities. Instead, we argue that individual differences in sensitivity to distributional regularities, as measured by tasks such as AGL, may be closely linked to, and possibly build on, other cognitive capacities, particularly beyond infancy. Several studies have indicated that performance on statistical learning tasks often draws on attentional resources, verbal working memory, and cognitive control (Frost et al., 2015; Kidd & Arciuli, 2016; Kidd et al., 2023; Siegelman, Bogaerts, Christiansen, & Frost, 2017). These influences may become more pronounced in older children and adults, who have increasingly powerful attentional and control capacities and may rely more on rehearsal and cognitive control during statistical learning. As a result, statistical learning performance in these groups likely reflects a complex interaction between sensitivity to structure and other supporting cognitive systems, for example, working memory (Conway, 2020).

Our own data are consistent with this. The *statistical learning: AGL offline* index was positively associated with all short‐term and working memory indices, most clearly with *forward digit span* and *n‐back*. Part of this association may arise at the point of knowledge expression rather than during learning itself: Tasks that require both the extraction of regularities and the subsequent retrieval of what has been learned draw on memory at retrieval as well as at encoding. This is not necessarily specific to offline statistical learning tests, as memory demands may be at work whenever distributional knowledge is deployed in everyday comprehension and production tasks.

Although our data support positive associations between statistical learning and executive functions, including working memory, recent work suggests that they may interact competitively. For example, Pedraza et al. (2024) and Smalle, Karantinou, and Möttönen (2026) reported negative associations between performance in statistical learning and executive function measures, while Smalle, Daikoku, Szmalec, Duyck, and Möttönen (2022) showed that statistical learning in speech segmentation can be boosted by a temporary depletion of the frontal cognitive control system in the brain. In contrast, other studies have reported positive relationships between statistical learning tasks and executive function measures (e.g., Lukács et al., 2026). Evidence from children is similarly mixed: Kidd et al. (2023) found positive or nonsignificant associations between visual and speech segmentation tasks and working memory in 6–7‐year‐olds. Taken together, these findings suggest that the relationship between statistical learning and executive functions is not uniform. Depending on task characteristics and the specific statistical learning and executive processes involved, executive functions may either support or constrain statistical learning, and developmental changes in cognitive control may further modulate this relationship (Smalle & Möttönen, 2023). These considerations highlight the importance of treating statistical learning as a multifaceted ability and of interpreting associations with other cognitive skills in light of developmental and methodological factors.

We also tested the strength of the relationship between cognitive skills and language across neurodevelopmental groups. The lack of detectable differences across TD, DLD, ADHD, and ASD groups found in correlation analyses suggests that the pattern of relationship between cognitive abilities and language is similar in different neurodevelopmental conditions. This may reflect a broader underlying pattern: that language development, whether typical or atypical, is supported by similar cognitive mechanisms across individuals. This possibility is consistent with dimensional accounts of neurodevelopment, which propose that core cognitive abilities such as working memory and sensitivity to input regularities contribute to language development in a graded rather than categorically distinct manner (e.g., Bishop, 2006; McGrath, Peterson, & Pennington, 2020). From this perspective, differences in language outcome arise not from qualitatively different processes across diagnostic groups but from quantitative variation in the efficiency or integrity of the underlying cognitive mechanisms. This interpretation aligns with previous research on statistical learning in neurodevelopmental disorders, which has yielded inconsistent results. Some studies have reported impairments in statistical learning in groups with DLD or ASD (e.g., Evans et al., 2009; Haebig et al., 2017; Lukács & Kemény, 2014), whereas others have found relatively intact performance, particularly when tasks minimize cognitive load or increase perceptual clarity (e.g., Jeste et al., 2015). These findings suggest that performance on statistical learning tasks is shaped by both individual cognitive profiles and specific task characteristics, rather than serving as a consistent marker of any particular condition, and point to the fact that individuals within the same diagnostic category often differ widely in both statistical learning and language skills (Arciuli & Conway, 2018). Our findings also align with recent calls for a more precise characterization of statistical learning in research on language impairments. As noted by Bogaerts et al. (2021), statistical learning tasks differ markedly in their computational demands, and performance reflects multiple interacting processes rather than a single unified mechanism. This also helps explain the variability of results reported across studies and across diagnostic groups.

Taken together with our results, this supports the idea that individual differences in statistical learning and related cognitive abilities may influence language development across diagnostic groups but do not uniquely define any single disorder. Supplementary analyses further support this interpretation: Although our study did not primarily aim to compare neurodevelopmental groups, we found no robust group differences in the pattern of language and cognitive abilities. There were some exceptions, with ASD being the strongest diagnostic group predictor of expressive vocabulary and DLD of sentence repetition, as expected. However, performance across groups was variable and partly overlapping, which reinforces the view that these neurodevelopmental profiles are best understood as dimensional rather than categorical, at least with respect to the assessed language abilities.

While the overall findings highlight a shared cognitive basis for language abilities across neurodevelopmental conditions, subtle descriptive differences between groups were also observed. Correlation estimates indicated that auditory perceptual speed and digit span showed slightly different associations with language measures in the ASD and DLD groups, compared to the others. The weaker correlations between digit span and language in the DLD group may simply reflect low variability in digit span scores. Regarding auditory perceptual speed, previous research indicates that atypicalities in low‐level auditory perception have been reported in both ASD and DLD, although not all individuals show such difficulties. In children who do, these perceptual differences have been linked to aspects of language development, but the strength and consistency of this association remains debated (Alcántara, Weisblatt, Moore, & Bolton, 2004; Arnett et al., 2018; Bishop, Carlyon, Deeks, & Bishop, 1999; Haesen, Boets, & Wagemans, 2011; McArthur & Bishop, 2004; Rosen, 2003; Tallal, 1976; Tallal & Piercy, 1973). The stronger associations observed between auditory perceptual speed and language performance in these groups may therefore reflect a greater reliance on bottom‐up perceptual processes in language acquisition and processing. This may be a result of weak linguistic representations in DLD (Joanisse & Seidenberg, 1998; Leonard, 2014; McGregor, Oleson, Bahnsen, & Duff, 2013) and a less efficient reliance on context in ASD (Frith & Happé, 1994; Happé, 1993). Nonetheless, given the overlap in correlation estimates across groups and the limited sample size, these descriptive patterns should be interpreted with caution. And even if more complex, condition‐specific relationships exist, the main conclusion holds: Domain‐general cognitive factors, especially working memory, seem to predict language abilities in similar ways across neurodevelopmental profiles.

A key limitation in our study is sample size within the neurodevelopmental groups. Although recruiting multiple neurodevelopmental groups within a single, well‐characterized sample is a strength of the study, the modest number of participants in each group limits statistical power to detect subtle group‐specific patterns or interactions. Accordingly, any similarities or differences between groups should be interpreted with caution, and confirming or refuting subtle group‐specific effects will require larger samples.

The scope of the language and statistical learning measures used is another limitation. We assessed language with two expressive tasks, picture naming and sentence repetition, and did not include direct measures of receptive vocabulary or grammatical comprehension. Our conclusions therefore apply specifically to expressive vocabulary and morphosyntactic production and may not extend to receptive language skills. Future work should include a broader range of expressive and receptive measures to determine whether the pattern of predictors observed here holds across different areas of language. Statistical learning was also assessed using a single task. AGL paradigms are widely used to study sensitivity to structured sequential regularities using both lower‐order, adjacent, and higher‐order, non‐adjacent statistical dependencies. We made this choice with the aim of capturing the complexity of patterns present in language, with particular regard to grammatical abilities measured by the *Sentence Repetition* task, as opposed to the widely used segmentation tasks, which operate only with adjacent dependencies. However, as these different statistical learning tasks capture partially distinct processes (Arciuli, 2017; Bogaerts et al., 2021), we could only cover part of statistical learning abilities. This also makes comparison with previous literature difficult because most child studies relied on the segmentation task (see Boeve et al., 2024). Including multiple paradigms would allow a more robust assessment of individual differences and help determine whether the observed relationships generalize across different forms of statistical learning and improve comparability with previous studies.

Finally, recent work has drawn attention to the reliability challenges in statistical learning tasks, which limit the extent to which stable individual differences can be captured (Arnon, 2020; Bogaerts et al., 2022; Siegelman, Bogaerts, & Frost, 2017). As lower reliability increases measurement noise and attenuates correlations with other variables, this broader methodological issue may explain why statistical learning accounted for a smaller proportion of the variance in language outcomes, compared to other cognitive measures.

## 5 Conclusion

This study explored the role of statistical learning in predicting vocabulary and grammatical abilities across typically developing children and those with DLD, ASD, and ADHD. Although statistical learning also predicted language performance, verbal working memory emerged as the strongest predictor of language abilities, emphasizing its centrality in language development across neurodevelopmental profiles. However, statistical learning indices were considerably less reliable than the other cognitive predictor indices, so associations involving statistical learning are likely to be underestimated. These results replicate and extend previous findings by demonstrating that individual differences in statistical learning contribute to language abilities not only in typically developing but also in atypical populations. They also point out that the contribution of individual differences in statistical learning might be, at least partly, explained by individual differences in working memory capacity.

Furthermore, we found no evidence that the relationships between cognitive abilities and language performance differed across diagnostic groups. These findings help place language development in a dimensional account of neurodevelopmental conditions: Language abilities do not appear to be qualitatively different in different conditions but appear to emerge from varying constellations of cognitive abilities. Overlapping cognitive profiles between diagnostic groups explain overlapping linguistic abilities and the absence of clear‐cut differences between the conditions.

## Conflict of Interest Statement

The authors declare no conflict of interest.

## Permission to Reproduce Material from Other Sources

The authors declare that they did not use any materials for the study that would have required permission for reproduction.

## Supporting information




**Appendix**: cogs70261‐supp‐0001‐SuppMat.pdf

## Data Availability

A comprehensive description of the tasks, indices, data analysis and results together with the data are provided in the Supporting Information, openly available here: https://doi.org/10.17605/OSF.IO/3V7NA.

## Note

1 Relatedly, throughout the article, we use the term domain‐general in the weak sense: We assume that statistical learning is present outside the domain of language, but we do not claim that a unitary statistical learning mechanism would underlie performance across modalities and stimulus types.
