# Supplementary material for: Re‐Evaluating the Role of Statistical Learning: Domain‐General Predictors of Language in Typical and Atypical Development
Source: Cogn Sci. 2026 Sep 9;50(9):e70261. doi: 10.1111/cogs.70261 (PMC13558848; doi:10.1111/cogs.70261)

A1. Method

A1.1. Exclusion criteria for the artificial grammar learning task

For the artificial grammar learning task, attention and task compliance in the online training phase were assessed using a Fisher's exact test. For each participant, we constructed a 2×2 contingency table comparing responses to target trials (hits and misses) and non-target trials (false alarms and correct rejections). Under the null hypothesis, responses were assumed to be independent of stimulus type, corresponding to equal response proportions for target and non-target trials. Participants were retained for further analyses if the proportion of responses to target words was significantly higher than the proportion of responses to non-target words ($p < .05$). Participants who did not meet this criterion were excluded, as their response patterns indicated insufficient attention to the task or non-compliance with instructions. No participants were excluded based on this criterion.

A1.2. Data analysis

We fit the xgboost models using grid search over the hyperparameters shown in Table 1.

Table 1

Hyperparameters of the XGBoost models

**hyperparameter**	**values in grid search**
number of trees	[50, 100]
model learning rate	[0.01, 0.05, 0.1]
maximum tree depth	[2, 3]
proportion of observations	[0.8, 1.0]
proportion of predictors	[0.2, 0.5, 0.8, 1.0]
minimum child weight	[1, 3, 5]

A2. Supplementary analyses

Missing values in the data are illustrated in Figure 1.

Figure 1

Missing observations in the data

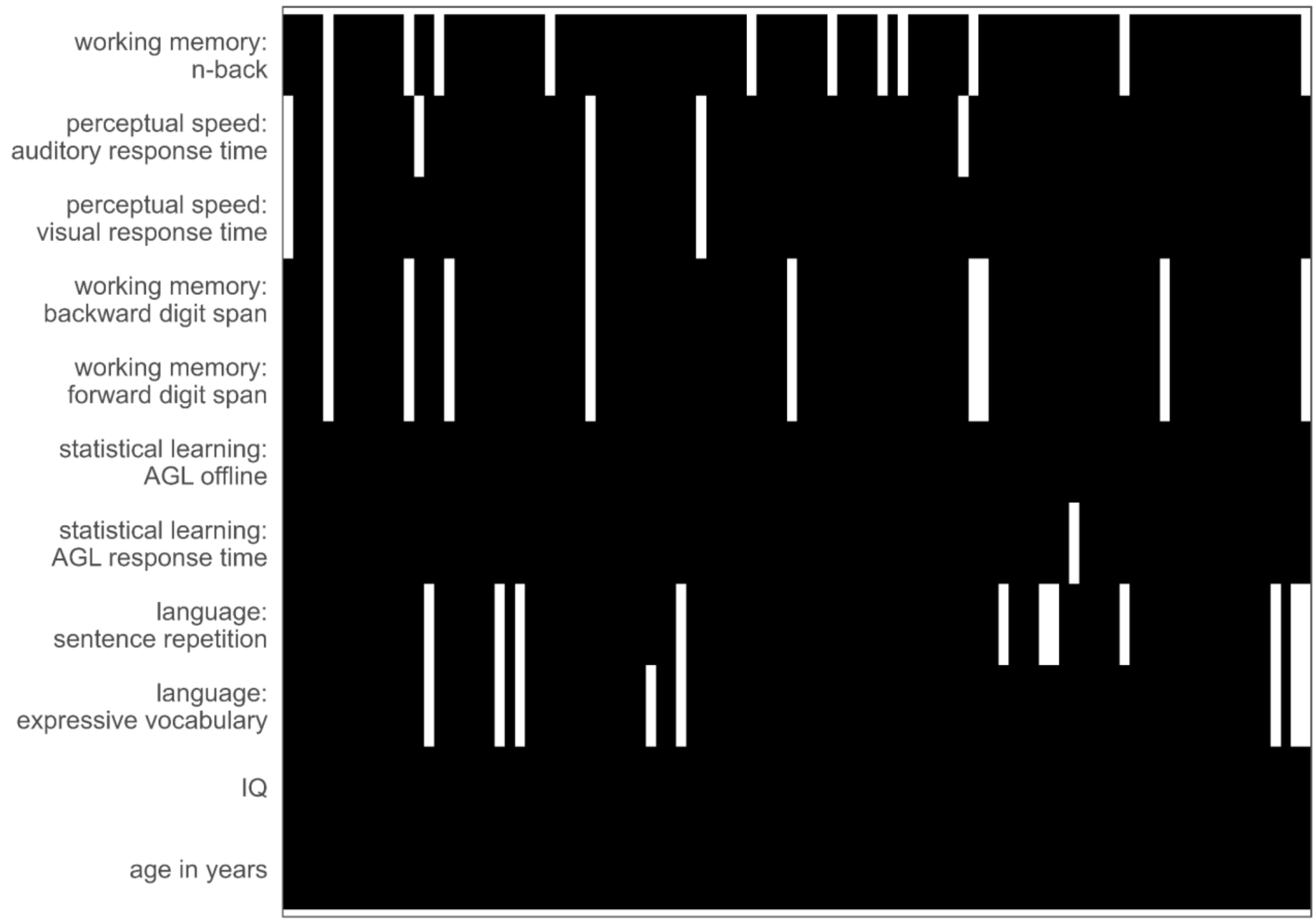

Note. Black cells indicate existing data, and white cells indicate missing data. Each column illustrates one participant.

We had complete observations for 71 children (per group: TD, 24; ADHD, 18; ASD, 16; DLD, 13).

For a better understanding of the data, we conducted supplementary analyses to test group differences in the case of all language and cognitive task scores. For each test, we predicted task scores from *neurodevelopmental group*. We found significant group differences in the case of *language: expressive vocabulary*, *language: sentence repetition*, *working memory: forward*

digit span, *working memory: backward digit span*. The detailed results of the analyses are summarised in Table 2.

Table 2

Group differences in task indices

model	group	beta	SE	t value	p value
age (years)	ADHD	0.26	0.48	0.54	0.591
age (years)	ASD	-0.38	0.47	-0.80	0.425
age (years)	DLD	0.32	0.51	0.64	0.525
IQ	ADHD	2.97	3.58	0.83	0.409
IQ	ASD	4.28	3.50	1.22	0.225
IQ	DLD	0.67	3.78	0.18	0.860
AGL reaction time difference	ADHD	-0.04	0.03	-1.29	0.200
AGL reaction time difference	ASD	-0.04	0.03	-1.48	0.142
AGL reaction time difference	DLD	-0.01	0.03	-0.24	0.813
AGL offline	ADHD	-0.01	0.02	-0.50	0.620
AGL offline	ASD	-0.03	0.02	-1.30	0.197
AGL offline	DLD	-0.03	0.03	-1.33	0.188
auditory reaction time	ADHD	0.02	0.02	0.90	0.371
auditory reaction time	ASD	0.04	0.02	1.83	0.071
auditory reaction time	DLD	0.03	0.03	1.00	0.319
visual reaction time	ADHD	0.03	0.03	0.89	0.374
visual reaction time	ASD	0.04	0.03	1.43	0.157
visual reaction time	DLD	0.05	0.03	1.51	0.134
backward digit span	ADHD	-0.77	0.33	-2.38	0.019
backward digit span	ASD	-0.64	0.32	-2.01	0.047
backward digit span	DLD	-0.92	0.34	-2.65	0.009
forward digit span	ADHD	-0.50	0.31	-1.63	0.107
forward digit span	ASD	-0.62	0.30	-2.06	0.043
forward digit span	DLD	-1.08	0.33	-3.29	0.001
n-back	ADHD	4.16	5.52	0.75	0.454

n-back	ASD	-8.16	5.59	-1.46	0.148
n-back	DLD	-4.05	6.04	-0.67	0.505
expressive vocabulary	ADHD	-0.03	0.03	-1.13	0.260
expressive vocabulary	ASD	-0.13	0.03	-5.03	<0.001
expressive vocabulary	DLD	-0.04	0.03	-1.31	0.192
sentence repetition	ADHD	-0.09	0.05	-1.98	0.051
sentence repetition	ASD	-0.18	0.05	-3.83	<0.001
sentence repetition	DLD	-0.26	0.05	-4.99	<0.001

Note. In each model, the TD group was the reference group, and statistics show the difference from the TD reference level. Significance values were not corrected for multiple testing.

The relationship between cognitive predictors and language task scores are illustrated in Figure 2 (*expressive vocabulary*) and Figure 3 (*sentence repetition*).

Figure 2

Relationship between expressive vocabulary and cognitive predictors across the neurodevelopmental groups

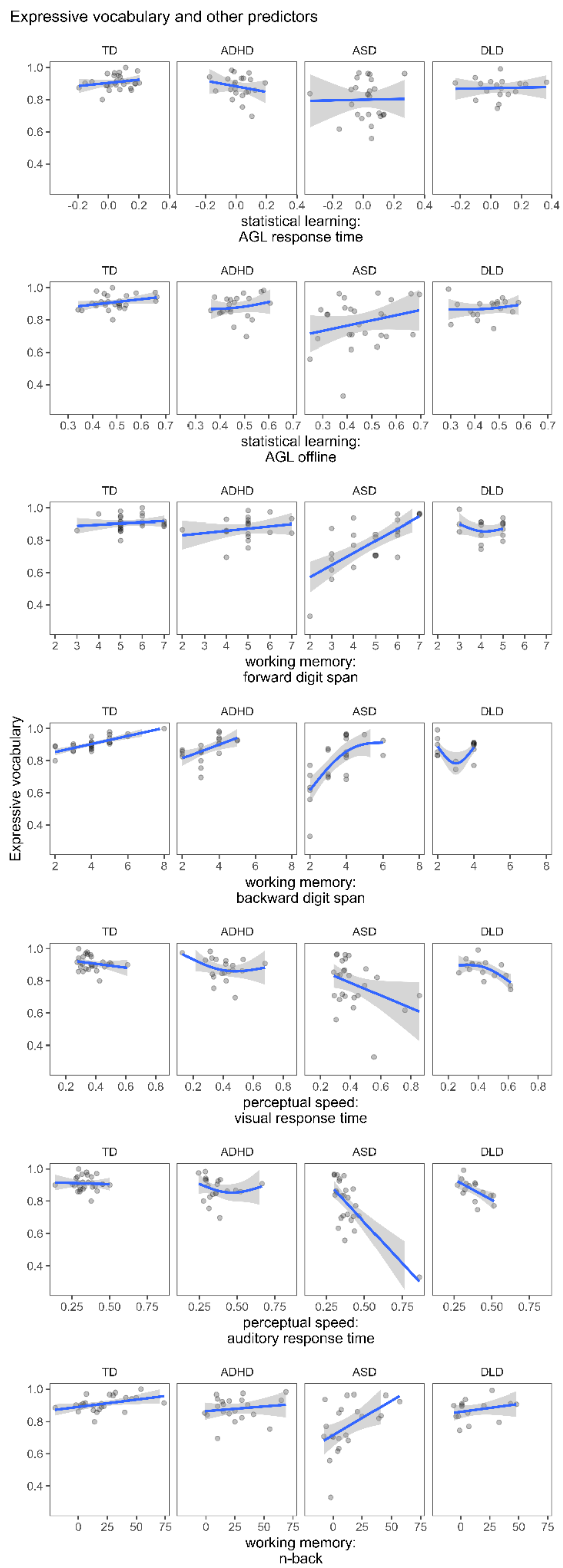

Figure 3

Relationship between sentence repetition and cognitive predictors across the neurodevelopmental groups

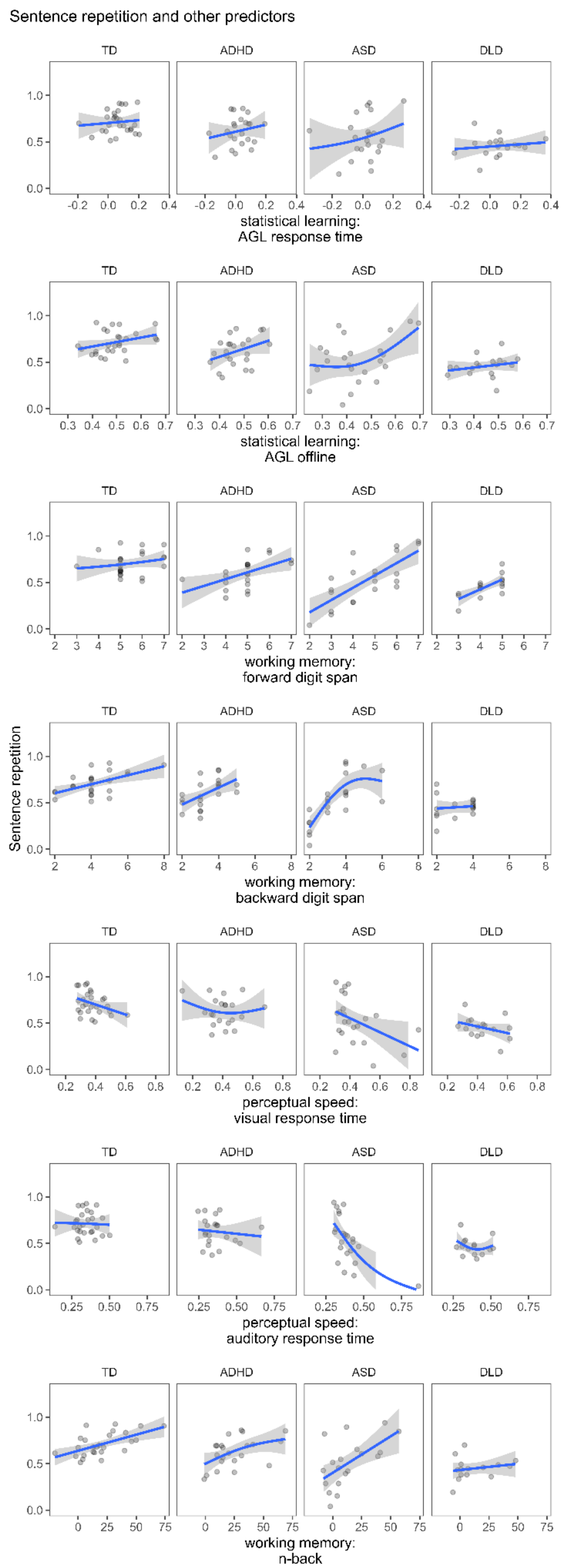

Supplement: Supplementary file 1 [file COGS-50-e70261-s001.pdf]
